# The Plant Decapeptide OSIP108 Can Alleviate Mitochondrial Dysfunction Induced by Cisplatin in Human Cells

**DOI:** 10.3390/molecules190915088

**Published:** 2014-09-19

**Authors:** Pieter Spincemaille, Hamed Alborzinia, Jeroen Dekervel, Petra Windmolders, Jos van Pelt, David Cassiman, Olivier Cheneval, David J. Craik, Julia Schur, Ingo Ott, Stefan Wölfl, Bruno P. A. Cammue, Karin Thevissen

**Affiliations:** 1 Centre of Microbial and Plant Genetics (CMPG), KU Leuven, Kasteelpark Arenberg 20, Heverlee 3001, Belgium; 2 Institute of Pharmacy and Molecular Biotechnology, Heidelberg University, Im Neuenheimer Feld 364, Heidelberg 69120, Germany; 3 Department of Hepatology and Metabolic Center, University Hospital Gasthuisberg, Herestraat 49, Leuven 3000, Belgium; 4 Division of Chemistry and Structural Biology, Institute for Molecular Bioscience, University of Queensland, Brisbane, Old 4072, Australia; 5 Institute of Medicinal and Pharmaceutical Chemistry, Technische Universität, Braunschweig, Beethovenstrasse 55, Braunschweig 38106, Germany; 6 Department of Plant Systems Biology, VIB, Technologiepark 927, Ghent 9052, Belgium

**Keywords:** cisplatin, OSIP108, glycolysis, respiration, real-time online monitoring

## Abstract

We investigated the effect of the *Arabidopsis thaliana*-derived decapeptide OSIP108 on human cell tolerance to the chemotherapeutic agent cisplatin (Cp), which induces apoptosis and mitochondrial dysfunction. We found that OSIP108 increases the tolerance of HepG2 cells to Cp and prevents Cp-induced changes in basic cellular metabolism. More specifically, we demonstrate that OSIP108 reduces Cp-induced inhibition of respiration, decreases glycolysis and prevents Cp-uptake in HepG2 cells. Apart from its protective action against Cp in human cells, OSIP108 also increases the yeast *Saccharomyces cerevisiae* tolerance to Cp. A limited yeast-based study of OSIP108 analogs showed that cyclization does not severely affect its activity, which was further confirmed in HepG2 cells. Furthermore, the similarity in the activity of the d-stereoisomer (mirror image) form of OSIP108 with the l-stereoisomer suggests that its mode of action does not involve binding to a stereospecific receptor. In addition, as OSIP108 decreases Cp uptake in HepG2 cells and the anti-Cp activity of OSIP108 analogs without free cysteine is reduced, OSIP108 seems to protect against Cp-induced toxicity only partly via complexation. Taken together, our data indicate that OSIP108 and its cyclic derivatives can protect against Cp-induced toxicity and, thus, show potential as treatment options for mitochondrial dysfunction- and apoptosis-related conditions.

## 1. Introduction

Cisplatin (*cis-*diamminedichloroplatinum (II), Cp) is one of the most widely used and effective chemotherapeutic agents and has been used to treat various types of cancer including lung, ovary and bladder cancer [1]. However, cytotoxic side effects [2] and cellular resistance to Cp have been reported [3,4]. The molecular mechanism underlying Cp-induced cell death is thought to arise from cross-linking of DNA, which results in cell-cycle arrest and apoptosis [5]. Moreover, Cp activates acid sphingomyelinase (aSMase) [6], leading to increased production of the apoptosis-inducer ceramide [7]. In addition, several studies have described a damaging effect of Cp on mitochondria in Cp treated cells [8]. For instance, the activity of complexes I to IV of the respiratory chain decrease upon Cp treatment [9]. As respiration of isolated mitochondria seems not inhibited by Cp, the latter indicates that Cp is probably not a direct inhibitor of complexes I to IV [10]. More recently, Cp has been shown to induce renal oxidative stress and to affect mitochondrial structure and function, whereas mitochondria-targeted anti-oxidants protect against Cp-mediated effects [11]. Moreover, Cp induces a mitochondria-dependent increase in reactive oxygen species (ROS) production, which significantly contributes to Cp-induced DNA damage-related cytotoxicity in prostate cancer cells [12].

We previously identified a bioactive *Arabidopsis thaliana*-derived decapeptide termed OSIP108 (Oxidative Stress-Induced Peptide 108) upon treatment of plants with the ROS-inducing herbicide paraquat (PQ) [13,14]. In addition, OSIP108 increases plant and yeast tolerance to oxidative stress-inducing agents like PQ and hydrogen peroxide, respectively [13]. Furthermore, OSIP108 prevents copper-induced (Cu) apoptosis in yeast and human cells [15]. Cu-induced toxicity is fundamentally linked to oxidative stress and apoptosis [16,17,18,19,20], and is implicated in the human pathology Wilson disease. The latter is characterized by excess Cu accumulation in the liver, resulting in acute liver failure or cirrhosis, as well as neurodegeneration due to elevated intracellular Cu levels [21,22,23,24]. Interestingly, OSIP108 also prevents Cu-induced cell death of neuroglioblastoma cells, as well as Cu-induced changes in liver morphology and oxidative stress levels in zebrafish larvae [25]. Like Cp, Cu induces apoptosis in human cells via activation of aSMase [16] and mitochondrial dysfunction [20]. For instance, Cu causes mitochondrial dysfunction by inducing oxidative stress [17,18,19] and by causing a deficiency of complex IV of the mitochondrial respiratory chain [26]. Thus, given the similarities in Cu and Cp-induced toxicity, in the present study we assessed the response of basic cellular metabolism of HepG2 cells upon treatment with the chemotherapeutic agent Cp and investigated the effect of OSIP108 on HepG2 tolerance to Cp. In addition, we analyzed the effect of OSIP108 analogs on Cp-stressed *Saccharomyces cerevisiae* cells and translated these results to HepG2 cells. All data indicate that OSIP108 can alleviate Cp-induced toxicity in yeast and human cells.

## 2. Results and Discussion

### 2.1. OSIP108 Increases Tolerance of the Human Hepatocyte HepG2 Model Cell Line to Cp

**Figure 1:**
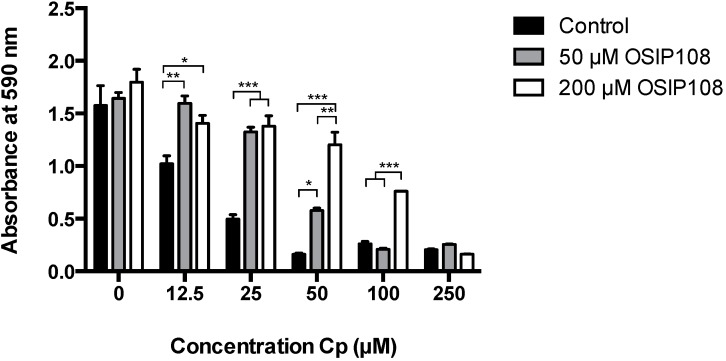
OSIP108 increases tolerance of HepG2 cells to Cp. HepG2 cells were incubated with control (1% DMSO, black bars) or OSIP108 (50 µM, grey bars or 200 µM, white bars) in absence (0 µM) or presence of low to high doses of Cp (12.5 µM–250 µM). Following 72 h incubation, cell viability was determined by MTT assay. All experiments were performed in triplicate and were repeated with different cell batches. (* *p <* 0.05; ** *p* < 0.01; *** *p* < 0.001; ANOVA test using Tukey correction).

We first investigated the effect of OSIP108 against Cp-induced toxicity in HepG2 cells. To this end, HepG2 cells were treated with various Cp concentrations (12.5 µM–250 µM) or control (0.9% NaCl, 0 µM Cp) in the presence of control (1% DMSO) or OSIP108 (50 µM or 200 µM in 1% DMSO) for 72 h, after which cell viability was determined by a 3-(4,5-dimethylthiazol-2-yl)-2,5-diphenyltetrazolium bromide (MTT) assay. As expected, a dose-dependent decrease in HepG2 cell viability was observed with increasing Cp concentrations, reaching maximal inhibition of cell viability at 50 µM Cp (Figure 1). Treatment with different OSIP108 concentrations (50 or 200 µM) of Cp-treated HepG2 cells (25 µM–50 µM) increased cell viability by at least 50%. Cell viability of HepG2 cells treated with a higher Cp dose (100 µM) could be increased by coincubation with 200 µM OSIP108. At Cp doses higher than 100 µM, OSIP108 could not protect HepG2 cells from Cp-induced cell death. These data indicate that OSIP108 increases tolerance of HepG2 cells to Cp.

### 2.2. OSIP108 Reduces Cp-Induced Inhibition of Respiration

**Figure 2:**
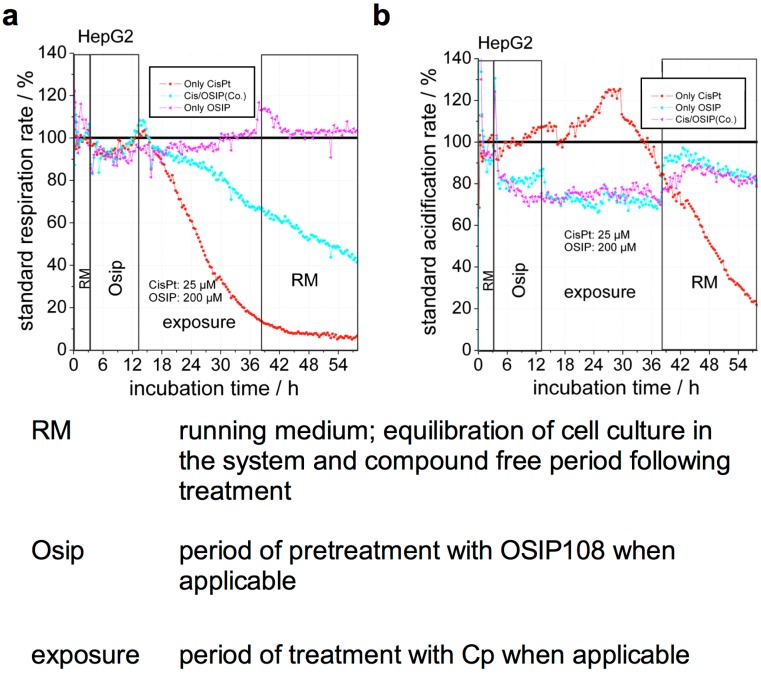
OSIP108 prevents Cp-induced respiration inhibition and decreases glycolysis in the human hepatoma HepG2 cell line. HepG2 cells were incubated with 25 µM Cp in presence of 200 µM OSIP108 or control (1% DMSO) using an exposure protocol with OSIP108 or control pretreatment in a Bionas 2500 cell biosensor chip system. **RM**: running medium, equilibration of cell culture in the system and compound free period following treatment; **Osip**: period of pretreatment with OSIP108 when applicable; **exposure**: period of treatment with Cp when applicable. Standard respiration rates (**a**) and acidification rates (**b**) were continuously monitored and are presented as percent activity relative to untreated control. The graphs show the measurements of one experimental run with 6 samples continuously analyzed in parallel; representative of three independent experiments.

To gain more insight into the protective effect of OSIP108 against Cp-induced cytotoxicity, we measured in real-time basic cellular metabolism of HepG2 cells using a Bionas 2500 cell biosensor chip. The latter measures changes in acidification and oxygen content of the medium, as an indirect measure of glycolysis and respiration, respectively [27]. Thus, we indirectly analyzed glycolysis and respiration in HepG2 cells during treatment with 25 µM Cp and 200 µM OSIP108, alone or in combination [10]. Upon treatment with Cp, we observed that respiration is immediately decreased (Figure 2a), whereas glycolysis is increased in the earlier phase of treatment. The extent of these effects varied between experimental repetitions, however, these effects rapidly decreased after about 15–17 h, marking a rapid onset of cell death at this time (Figure 2b). Although OSIP108 treatment did not affect respiration (Figure 2a), we observed that OSIP108 treatment reduced the glycolytic rate by approximately 30% and this effect was abrogated upon removal of OSIP108 (Figure 2b). Co-treatment with OSIP108 and Cp following pre-incubation with OSIP108 significantly reduced the Cp-mediated decrease in respiration (Figure 2a), whereas the effect on glycolysis of the combination of Cp and OSIP108 as compared to OSIP108 was similar (Figure 2b). These data indicate that OSIP108 protects cells against Cp-induced inhibition of respiration and affects the rate of glycolysis in HepG2 cells.

Note that despite the metabolic shift of cancerous cell lines from aerobic respiration to glycolysis, also known as the Warburg effect [28,29], which does not reflect normal mitochondrial activity in normal mammalian cells, immortalized cell lines are still routinely used to study toxicity mechanisms [30,31,32,33,34]. In addition, the Bionas 2500 sensor system has already been used to study the effect of several routinely-used hepatotoxic drugs on HepG2 cells with focus on mitochondrial function [30]. Hence, to further explore the biological mechanism underlying the beneficial effect of OSIP108 on Cp-induced toxicity in HepG2 cells, we analyzed basic cellular metabolism upon Cp and/or OSIP108 treatment. Strikingly, treatment with OSIP108 alone leads to a clear reduction of glycolysis, which remains reduced even after addition of Cp in cells pretreated with OSIP108 at amounts exceeding those of Cp (molar ratio ≥ 8). In addition, OSIP108-mediated decreased glycolysis is restored upon removal of OSIP108, implicating a direct effect of OSIP108 on cellular metabolism. We previously reported that OSIP108 perturbs homeostasis of sphingolipids [15], a class of lipid molecules that have a pivotal role as membrane constituents and as signaling molecules orchestrating cellular processes, such as inflammation, apoptosis and mitochondrial function [35,36,37,38,39,40]. It is, therefore, plausible that OSIP108-induced aberrancies in SL homeostasis affect the rate of glycolysis in HepG2 cells. However, given the aberrant cellular metabolism of cancerous cell lines, further research is needed to determine whether OSIP108 induces similar metabolic changes in normal cell lines.

Additionally, whereas treatment with Cp led to an immediate decrease in respiration, the latter was not affected by OSIP108 in absence of Cp. Nonetheless, co-treatment of Cp-treated cells with excess OSIP108 clearly reduced but did not abolish the strong inhibitory effect of Cp on respiration. Interestingly, this observed reduction in respiration inhibition resembles decreased respiration rates after treatment with lower amounts of Cp (data not shown), suggesting reduced cellular Cp uptake in presence of OSIP108.

**Figure 3:**
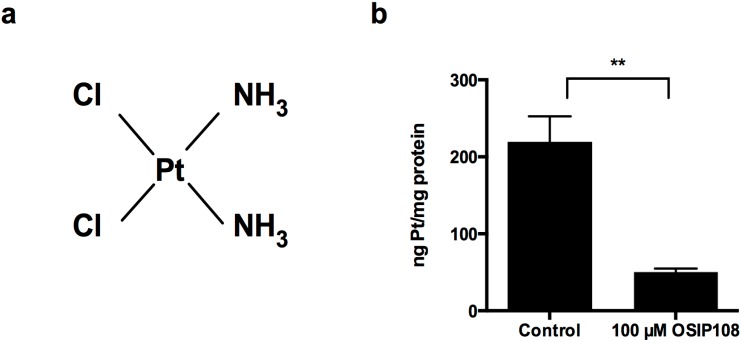
OSIP108 blocks uptake of Cp in HepG2 cells (**a**) Chemical structure of Cp; (**b**) HepG2 cells were exposed to Cp (10 µM) for 48 h in absence (control, 1% DMSO) or presence of OSIP108 (100 µM) and intracellular platinum (Pt) was measured by HR-CS AAS. Results are given at ng Pt per mg of protein in the cell lysates. Data is average from three independent experiments. (** *p <* 0.01; Student *t*-test).

### 2.3. OSIP108 Affects Cp Uptake in HepG2 Cells

As the activity of Cp depends directly on the efficiency of the cellular uptake [41], as is the case for many other cytotoxic compounds, we analyzed whether OSIP108 influences Cp uptake in HepG2 cells. Given that Cp is a platinum-containing agent (Figure 3a), we determined platinum (Pt) levels in cell lysates of HepG2 cells treated with Cp in presence or absence of OSIP108. To this end, cells were incubated with 10 µM Cp in the absence (1% DMSO) or presence of 100 µM OSIP108 for 48 h. After removal of medium, cells were washed and lysed and the amount of intracellular Pt in the cell lysate was analyzed by high-resolution continuum source atomic absorption spectrometry (HR-CS AAS). We observed that Cp treatment (10 µM) resulted in approx. 200 ng Pt/mg protein present in the cell lysates (Figure 3b). In the presence of OSIP108 (100 µM) however, the amount of Pt was significantly reduced to 50 ng Pt/mg cellular protein. These data indicate that coincubation of cells with OSIP108 and Cp reduces Cp-uptake by 4-fold (Figure 3b). Hence, it seems that OSIP108 prevents Cp-uptake, leading to a decreased intracellular Cp concentration and thereby reduces Cp-induced toxicity in HepG2 cells.

### 2.4. Activity of OSIP108 Analogs on Cp-Stressed Yeast

**Figure 4:**
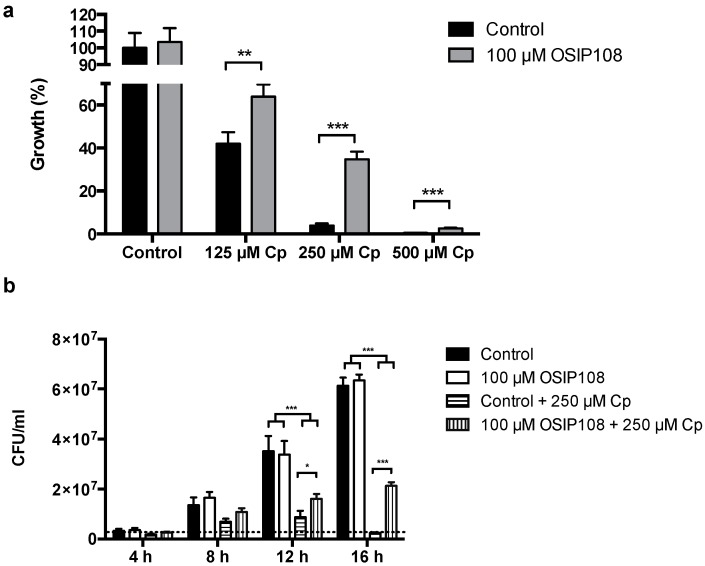
OSIP108 increases yeast tolerance to Cp. (**a**) WT yeast cells were incubated with Cp (125 µM–500 µM) for 16 h in presence of control treatment (2.5% DMSO, black bars) or 100 µM OSIP108 (grey bars). Growth was calculated by determining CFU/mL as compared to control treatment (100%). Experiment performed in quadruplicate, biological repeats is three (** *p* < 0.01; *** *p* < 0.001; Student *t*-test) (**b**) WT yeast cells were treated with control (2.5% DMSO; black bars and horizontal dashed bars) or 100 µM OSIP108 (white bars and vertical dashed bars) in absence (black and white bars) or presence (horizontal and vertical dashed bars) of 250 µM Cp. CFU/mL were determined periodically during 16 h of treatment. Dashed line indicates the average CFU/mL at the time point that the experiments were initiated. Biological repeats is three. (* *p* < 0.05; *** *p* < 0.001; ANOVA test using Tukey corrections).

To obtain more insight into the structure-activity relationships (SAR) of OSIP108, we assessed the effect of OSIP108 analogs on Cp-stressed *S. cerevisiae*. Using Cp-induced toxicity in yeast allows high-throughput analysis of the SAR of the peptide, as compared to an additional mammalian cell line, next to the aspect that pathways that, e.g., modulate mitochondrial function and apoptosis are well conserved [42,43]. In line with the HepG2 data, we observed a dose-dependent decrease in yeast growth upon treatment with Cp (125 µM–500 µM) (Figure 4a). Coincubation of the yeast cells with OSIP108 (100 µM) significantly increased yeast growth as compared to control (2.5% DMSO) (Figure 4a). As our results showed that 100 µM OSIP108 has the most pronounced effect on *S. cerevisiae* growth in the presence of 250 µM Cp, we further analyzed the effect of 250 µM Cp and control (2.5% DMSO) or 100 µM OSIP108 in a time-dependent manner and expressed it as colony forming units/mL (CFU/mL) as a measure for cell viability and cell growth. Cell growth was observed in absence of Cp starting from 8 h of incubation, which was similar for the OSIP108-treated cells (Figure 4b). In addition, low growth was observed for the Cp-treated cells after 8 h up to 12 h of incubation, after which the CFU/mL dropped, indicating cell death (Figure 4b). Conversely, OSIP108 significantly increased CFU/mL of Cp-treated cells after 12 h and 16 h of incubation. Hence, this indicates that 250 µM Cp inhibits cell growth, and this effect is abrogated upon treatment with OSIP108.

**Figure 5:**
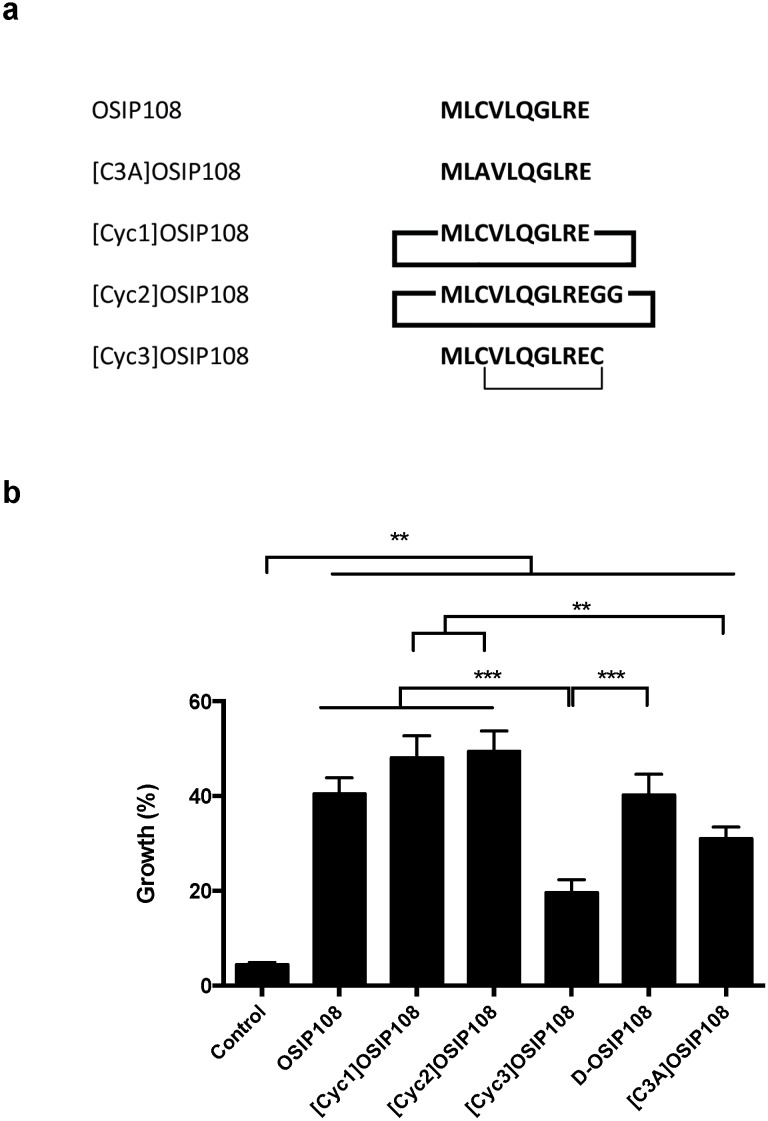
OSIP108 variants increase yeast tolerance to Cp. (**a**) Amino acid sequence and structure of OSIP108, [C3A]OSIP108 and cyclic derivatives tested in this study. Thick black lines represent head-to-tail cyclization; thin black line indicates disulfide bridge; (**b**) WT yeast cells were incubated with 250 µM Cp for 16 h in presence of control (2.5% DMSO) or 100 µM OSIP108, [Cyc1]OSIP108, [Cyc2]OSIP108, [Cyc3]OSIP108, d-OSIP108 or [C3A]OSIP108. Following incubation, cells were plated onto YPD agar plates and growth was calculated by determining CFU/mL as compared to cells receiving no Cp. Experiment performed in quadruplicate, biological repeats is three. (** *p* < 0.01; *** *p* < 0.001; ANOVA test using Tukey corrections).

Subsequently, the effect of 100 µM OSIP108 on yeast viability in presence of 250 µM Cp after 16 h of incubation was the most pronounced, and, thus, these conditions were chosen to evaluate the effect of OSIP108 analogs on yeast growth in presence of Cp. We investigated the effect of three cyclic OSIP108 derivatives (Figure 5a) on Cp-stressed yeast. Cyclotides, cyclic peptides found in plants [44], are exceptionally resistant to enzymatic degradation, which makes them useful scaffolds in peptide-based drug design [45]. To obtain the first cyclic derivative, termed [Cyc1]OSIP108, the N- and C-termini of the native OSIP108 peptide were joined head-to-tail to yield cyclo-MLCVLQGLRE. The second cyclic derivative ([Cyc2]OSIP108) was cyclized head-to-tail following addition of two glycine residues to the C-terminus of the native peptide (cyclo-MLCVLQGLREGG). The third cyclic OSIP108 form ([Cyc3]OSIP108) was synthesized by addition of a C-terminal cysteine residue in order to form a disulfide bridge with the cysteine at position 3; this led to the formation of a disulfide-cyclized peptide (MLCVLQGLREC (cyclo-C3-C11). An additional d-stereoisomer variant OSIP108 (d-OSIP108) was also included, allowing us to investigate any putative binding of OSIP108 with a receptor. The use of mirror image peptides is widely used as a tool to distinguish mechanisms of action that require binding to a stereospecific receptor (e.g., protein binding) from those that do not (e.g., chemical complex formation or binding to membranes) [46]. Additionally, d-stereoisomers are less sensitive to peptide proteases [47,48]. Formation of OSIP108 dimers via Cys-linkage of two OSIP108 monomers was excluded by chromatography or mass spectrometry [49]. Lastly, we included a [C3A]OSIP108 variant, as thiol group-containing agents are known to prevent Cp-induced toxicity by formation of thiol-Cp-thiol complexes [50].

We consistently found that the native peptide as well as all the tested peptide analogs significantly increased yeast growth in presence of Cp (Figure 5b). In addition, we observed that the activity of [Cyc3]OSIP108 was significantly lower than that of the native peptide, [Cyc1]OSIP108, [Cyc2]OSIP108 and d-OSIP108. Furthermore, the activity of [C3A]OSIP108 was significantly lower as compared to [Cyc1]OSIP108 and [Cyc2]OSIP108. As thiol group-containing agents are known to prevent Cp-induced toxicity by Cp complexation via formation of thiol-cisplatin-thiol complexes [50], the decrease in anti-Cp activity of [Cyc3]OSIP108 could be attributed to its inability to form complexes with Cp. Indeed, [C3A]OSIP108 showed significantly reduced activity as compared to [Cyc1]OSIP108, [Cyc2]OSIP108, and not the native peptide, suggesting that the latter two peptides exhibit improved activity as compared to the native peptide, and that the effect of OSIP108 against Cp-induced toxicity seems only partly attributed to Cp complexation. The fact that the mirror image form of OSIP108 is equipotent to the l-stereoisomer is consistent with this mechanism of action since complex formation would not be affected by the stereochemistry. In addition, as the [Cyc3]OSIP108 analog is supposedly unable to complexate Cp, this suggests that this type of cyclization imposes steric constraints on the peptide structure by interfering with the spatial organization of the amino acid sidechains resulting in additional loss of anti-Cp activity. These data indicate that OSIP108 probably does not interact with a specific receptor, as its d-stereoisomer is as active as native OSIP108, that cyclic peptide modifications do not severely affect its activity, unless the Cys^3^ is involved in thecyclization and that OSIP108 protects against Cp-induced toxicity at least partly by Cp complexation.

**Figure 6:**
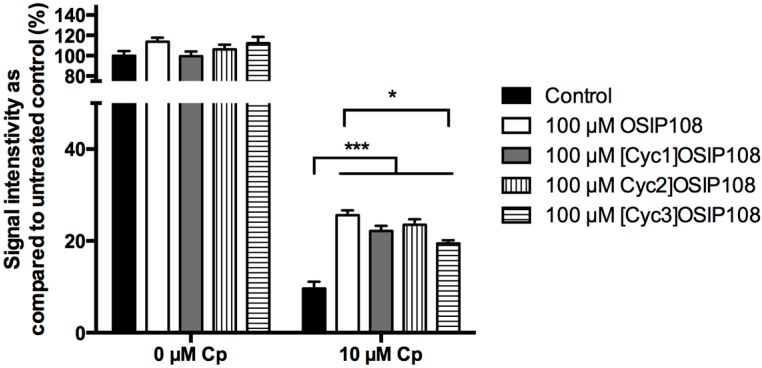
OSIP108 variants increase HepG2 tolerance to Cp. HepG2 cells were incubated with control (1% DMSO, black bars) or 100 µM OSIP108 (white bars), [Cyc1]OSIP108 (grey bars), [Cyc2]OSIP108 (vertical dashed bars) or [Cyc3]OSIP108 (horizontal dashed bars) in absence (0 µM Cp) or presence of 10 µM Cp. Following 72 h of incubation, cell proliferation was determined by BrdU assay and expressed as signal intensity as compared to untreated control. Technical repeats is three. (* *p* < 0.05; *** *p* < 0.001; ANOVA test using Tukey corrections).

To validate Cp-induced toxicity in *S. cerevisiae* as a model to perform SAR studies on OSIP108, we investigated the effects of native OSIP108, [Cyc1]OSIP108, [Cyc2]OSIP108 and [Cyc3]OSIP108 on Cp-induced toxicity in HepG2 cells by BrdU assay, as an alternative for MTT viability assay. The BrdU assay is based on the incorporation of the nucleoside analog 5-bromo-2'-deoxyuridine (BrdU) into replicating DNA and subsequent detection, and, thus, is a measure for cell proliferation. In a first step, HepG2 cells were treated with different Cp doses and cell viability and cell proliferation was determined by MTT assay and BrdU assay, respectively. While only marginal effects on cell viability were observed by MTT staining at low Cp doses (<12.5 µM Cp), we observed a dose-dependent decrease in cell proliferation by BrdU assay upon treatment with low Cp doses (data not shown), resulting in almost maximal inhibition of cell proliferation upon treatment with 10 µM Cp (Figure 6). These data indicate that Cp-induced decreased cell proliferation in HepG2 cells precedes Cp-induced decreases in cell viability. Indeed, Cp is known to induces cross-linking of DNA and thus inhibits cell replication and concomitantly triggers cell death [5]. Subsequently, we investigated the effect of 100 µM OSIP108 and the three cyclic analogs on cell proliferation upon treatment with 10 µM Cp. We observed that all tested peptides significantly increased cell proliferation in presence of Cp (Figure 6). Interestingly, the anti-Cp effect of [Cyc3]OSIP108, as measured by BrdU assay, was significantly lower as compared to the native peptide, as was also evident from our yeast data (Figure 5b). Taken together, this indicates that cyclic peptides can also increase HepG2 tolerance to Cp, and that Cp-induced toxicity in yeast can be used as a model to perform SAR studies on OSIP108 regarding the anti-Cp effect in a mammalian cell model.

Although OSIP108 strongly reduces Cp uptake in HepG2 cells, which is likely to be mediated by Cp complexation, we hypothesize that this is not the sole mechanism mediating the protective effect as OSIP108 also impacts on basic cellular metabolism (this study) and sphingolipid metabolism [15]. However, it cannot be excluded that Cp complexation significantly contributes to the protective effect of OSIP108 against Cp-induced toxicity; additional OSIP108-mediated effects on cellular metabolism might also play an important role. For instance, 200 µM OSIP108 increases HepG2 viability in presence of 50 µM Cp (1:4 Cp/OSIP108 ratio) to an extent that equates to residual viability of HepG2 cells treated with 12.5 µM Cp. This corresponds to 75% reduction in Cp uptake. Nevertheless, as a 1:10 Cp/OSIP108 ratio results in a 75% reduction of Cp uptake, a lower Cp/OSIP108 ratio could result in less pronounced reductions in Cp uptake.

## 3. Experimental Section

### 3.1. Materials and Microorganisms

The yeast strain used in this study was *Saccharomyces cerevisiae* wild type strain BY4741 (WT) and was cultured in SC (0.77 g/L complete amino acid supplement mixture (CSM) (Bio 101 Systems); 6.7 g/L yeast nitrogen base without amino acids (YNB); 20 g/L glucose). To determine colony-forming units (CFU/mL), cells were routinely plated on YPD agar plates (1% yeast extract, 2% peptone, 2% glucose, 1.5% agar). HepG2 cells (ATCC) were grown in DMEM (Gibco, Invitrogen, Carlsbad, CA, USA) supplemented with 10% FCS (PAA, Pasching, Austria) and 1% penicillin/streptomycin (Gibco Invitrogen) using standard tissue culture conditions. Running medium (RM) consisted of DMEM without carbonate buffer (PAN-Biotech GmbH, Aidenbach, Germany) supplemented with 1 g/L glucose, 0.1% FCS, 1 mM HEPES pH 7.4 and 1% penicillin/streptomycin. OSIP108 (MLCVLQGLRE, MW = 1161.43 g/mol) and [C3A]OSIP108 (MLAVLQGLRE, MW = 1129.38 g/mol) were purchased from Thermo Fisher Scientific (Ülm, Germany). Cyclic OSIP108 forms ([Cyc1]OSIP108 (cyclo-(MLCVLQGLRE), MW = 1143.42 g/mol), [Cyc2]OSIP108 (cyclo-(MLCVLQGLREGG), MW = 1257.52 g/mol)) and [Cyc3]OSIP108 (cyclo-C3-C11-(MLCVLQLGREC, MW = 1262.58 g/mol)), as well as d-OSIP108 (all d-amino acids) were synthesized as described [49]. *Cis*-diamminedichloroplatinum (II) (cisplatin, Cp) (Sigma-Aldrich, St. Louis, MO, USA) was dissolved in 0.9% NaCl or DMSO as indicated. Note that the OSIP108 rescuing effect on Cp-induced toxicity was apparent irrespective of the type of Cp solvent. As reported by Cunha and coworkers [51], variability in Cp cytotoxicity was inevitable between independent experiments. DMSO was used for solubilizing peptides.

### 3.2. Cp Toxicity in HepG2 Cells

HepG2 cells were seeded at a density of 5.000 cells/well in 96 well-plates for 24 h prior to treatment with 1% DMSO (control) or different OSIP108 concentrations (50 µM or 200 µM) in presence of 0.9% NaCl (control) or Cp (12.5µM–250 µM). Following 72 h of incubation, as measure for cell viability, 3-(4,5-dimethylthiazol-2-yl)-2,5-diphenyltetrazolium bromide (MTT) conversion was assessed according to the manufacturer’s instructions.

### 3.3. BrdU Assay in Cp-Treated HepG2 Cells

HepG2 cells were seeded at a density of 10.000 cells/well in 96-well plates prior to treatment with 1% DMSO (control) or 100 µM OSIP108, [Cyc1]OSIP108, [Cyc2]OSIP108 or [Cyc3]OSIP108 in the presence of 0.9% NaCl (control) or 10 µM Cp. Following 72 h of incubation, cell proliferation was assessed by using the Cell Proliferation ELISA BrdU (colorimetric) kit (Roche Diagnostics, Mannheim, Germany) according to the manufacturer’s instructions.

### 3.4. Real-Time Monitoring of HepG2 Metabolism

Real-time monitoring of toxicity and metabolic response to Cp treatment was performed using the microfluidic cell biosensor chip system BIONAS 2500 (Bionas, Rostock, Germany), recording changes in medium pH, as an indirect measure for rate of glycolysis, and oxygen, an indirect measure for respiration rate, as described earlier [10]. Parameters were continuously recorded, while medium conditions were maintained at defined values by medium exchange in 20 min cycles (4 min flow, 16 min stop). Before measurement cells were seeded at 20.000 cells/chip followed by overnight incubation in a standard tissue culture incubator, to establish the cell layer on the chip surface. Sensorchips were then transferred to the microfluidic biosensor chip workstation (Bionas 2500) and equilibrated in flow conditions for 4 h with RM before treatment started. Sensorchip cultures were subsequently pre-incubated with 1% DMSO (control) or 200 µM OSIP108 for 4 h before transfer into the microfluidic workstation, and then pre-incubated for an additional 10 h in the microfluidic system. Next, sensorchip cultures were treated with RM or 25 µM Cp in presence of 1% DMSO or 200 µM OSIP108 for 24 h, after which cells were cultured for an additional 24 h in drug-free RM.

### 3.5. Cellular Cp Uptake in HepG2 Cells

HepG2 cells were treated with control (1% DMSO) or 100 µM OSIP108 in presence of 10 µM Cp for 48 h as described in Section 3.2. Subsequently, cellular platinum (Pt) content was measured by high-resolution continuum source atomic absorption spectrometry (HR-CS AAS; contrAA^©^ 700, Analytik Jena AG). Pt was measured at 265.945 nm (emission line). For sample preparation, cells were harvested and cell lysates were prepared by 30 min of ultrasonication. Protein content of each sample was determined in a Bradford assay for normalization and matrix matched calibration. From each sample 25 µL aliquots were injected into coated standard graphite tubes (“AAS IC-Standardrohr, beschichtet”, AnalytikJena AG) and analyzed running the following temperature program: drying (i): Temp: 90 °C, Ramp: 6 °C·s^−1^, Hold: 15 s; drying (ii): Temp: 130 °C, Ramp: 10 °C·s^−1^, Hold: 30 s; drying (iii): Temp: 500 °C, Ramp: 50 °C·s^−1^, Hold: 30 s; pyrolysis: Temp: 1300 °C, Ramp: 300 °C·s^−1^, Hold: 10 s; AZ (zeroing): Temp: 1300 °C, Ramp: 0 °C·s^−1^, Hold: 5 s; atomization: Temp: 2300 °C, Ramp: 1500 °C·s^−1^, Hold: 8 s; tube cleaning: Temp: 2400 °C, Ramp: 500 °C·s^−1^, Hold: 4 s). During measurements, the graphite tube was purged with a constant argon gas flow, which was only interrupted during zeroing and atomization steps. Calibration for Pt quantification was done with Cp. Results were calculated as ng Pt per milligram of cellular protein.

### 3.6. Yeast Growth Assays

An overnight yeast culture in SC (average OD600 = 3.5) was diluted to OD600 = 0.1 in fresh SC and incubated with 2.5% DMSO (control) or Cp (125 µM–500 µM) for 16 h (30 °C, 250 rpm) in the presence of 2.5% DMSO (control) or 100 µM peptide. Following treatment, cells were plated onto YPD agar plates and percentage growth was calculated by determining CFU/mL as compared to cells receiving no Cp.

### 3.7. Statistical Analysis

Values are presented as mean with standard error (SEM). Data were analyzed by GraphPad Prism 6.0. *p* < 0.05 was considered as statistically significant.

## 4. Conclusions

In conclusion, our observations show that the plant-derived peptide OSIP108 or analogs increase yeast and human cell tolerance to Cp. As Cp induces apoptosis and mitochondrial dysfunction, our results suggest that OSIP108 and its cyclic derivates show promise as potential treatment options for mitochondrial dysfunction- and apoptosis-related conditions. Indeed, we recently showed that OSIP108 prevents Cu-induced apoptosis in yeast and human cells, as well as Cu-induced liver damage in a zebrafish model for the human pathology Wilson disease [15,25]. However, further research is needed to further unravel specific underlying mechanistic effects that are involved in the OSIP108-mediated protective effect against Cp-induced toxicity.
